# The Blood

**Published:** 1915-09

**Authors:** Charles W. Gould

**Affiliations:** Candler Building, Atlanta, Ga.


### Journal-Record of Medicine

Successor to Atlanta Medical and Surgical Journal, Established 1855
and Southern Medical Record, Established 1870
**OWNED BY THE ATLANTA MEDICAL JOURNAL COMPANY**
PUBLISHED MONTHLY

*Official Organ Fulton County Medical Society, State Examing Board, Atlanta, Birmingham and Atlantic Railroad Surgeon's Association, Chattahoochee Valley Medical and Surgical Association, Etc.*

RICHARD R. DALY, M. D., Editor
BERNARD WOLFF, M. D., Supervising Editor
A. W. STIRLING, M. D., C. M., D. P. H.; J. S. HURT, B. Ph., M.D.
GEO. M. NILES, M. D., W. J. LOVE, M. D., (Ala.); Associate Editors
E. W. ALLEN, Business Manager
**COLLABORATORS**
W. F. WESTMORELAND, M. D., General Surgery
F. W. McRAE, M. D., Abdominal Surgery
ALLEN H. BUNCE, Medical Societies
E. B. BLOCK, M. D., Diseases of the Nervous System
MICHAEL HOKE, M. D., Orthopedic Surgery
CYRUS W. STRICKLER, M. D., Legal Medicine and Medical Legislation
E. C. DAVIS, A. B., M. D., Obstetrics
E. G. JONES, A. B., M. D., Gynecology
ALLEN H. BUNCE, M. D., Pathology and Bacteriology
R. T. DORSEY, JR., B. S., M. D., Medicine
L. M. GAINES, A. B., M. D., Internal Medicine
GEO. C. MIZELL, M. D., Diseases of the Stomach and Intestines
L. B. CLARKE, M. D., Pediatrics
EDGAR PAULLIN, M. D., Opsonic Medicine
THEODORE TOEPEL, M. D., Mechano Therapy
R. R. DALY, M. D., Diseases of the Eye, Ear, Nose and Throat
BERNARD WOLFF, M. D., Diseases of the Skin
E. G. BALLENGER, M. D., Diseases of the Genito-Urinary Organs

VOL. LXII. ATLANTA, GA., SEPTEMBER, 1915. NO. 6

### THE BLOOD.

By CHARLES W. GOULD, M. D.

Blood has always been known as the most important of the body fluids, most important to the life of the individual.

Until the invention of the asphyxiating gas and the use of the submarines in warfare, weapons have all been designed to cause a sufficient amount of hemorrhage in the enemy to bring about his death.

So important is blood to life as recognized by the ancients, that they submitted it for the word life; as "his blood be on thy head." They were forbidden to eat blood for "the blood is the life."

It has always had a mysterious effect on the imagination of people.

It has appeared in literature, in Bluebeard and in Lady Macbeth, as being hard to remove.

It even affects cattle in a mysterious manner; an old "Bossy" catching the odor of freshly shed blood becomes metamorphosed into a raging terror.

A pregnant mare will abort her foal when frightened by fresh blood.

The blood of pure Castilian is supposed to have a different shade from the blood of those who have a trace of Moroccan blood, hence they used the term "Blue Blood," which now denotes aristocracy in general.

Wells says "the function of the blood is to maintain an equilibrium in the temperature, chemical composition and osmotic pressure between all parts of the body; it follows that it is never of exactly the same composition at any two places or at two different times."

The different tissue cells of the body are always giving off parts of their substance into the blood stream in the course of metabolism. So all the chemicals that enter into the formation of the different tissue cells may at times be found in the blood.

Besides the blood receives new substances into its composition from the food and drink ingested by the individual and from the air in the course of respiration.

Blood responds to stimuli of different kinds and makes innumerable substances by such response.

For instance: Blood stimulated by a poison gradually makes an anti-poison or anti-toxin. Bacteria irritating the blood, if they do not everwhelm, bring about the manufacture of bacteriolytic substances or anti-bodies which destroy them.

There are about ten to fourteen pounds of blood in the body, the red corpuscles make up 40 to 50 per cent. of this, the rest of the blood is Blood Plasma, Leukocytes and Platelets.

The red corpuscles are mostly made up of hemoglobin also containing globulin, nucleoprotein, lecithin, and cholesterin.

The hemoglobin is held in the cell by the stroma, by what process is not accurately known, some think by chemical combination, others by a sac-like membrane, or perhaps the stroma is a spongioplasm.

The giving up of this hemoglobin is called hemolysis and the principles of finding substances which will bring this about is used in the Wasserrmann Test, and other so-called complement fixation tests.

Hemolysis may be brought about by exposing the cells to distilled water, then the salts of the hemoglobin leave the stroma by osmosis; it is also done by heating and freezing, and by snake venom. Ammonia and urea cause hemolysis, while sugar and sodium chloride prevent it. The fact that cholesterin in excess prevents the hemolysis of the hemoglobin in the presence of Saponin suggests that it is the cholesterin which may be the part of the stroma affected.

Ether, chloroform, and Amyl Alcohol hemolyze blood and are also solvents of cholesterin.

Bile acid and salts bring on jaundice by hemolysis of the blood brought about by their action on the cholesterin and lecithin of the red corpuscles.

It has been strongly advocated lately that the amount of cholesterin in the blood of the patient has a great deal to do with positivity of the Wasserrman reaction and should therefore be taken into account when the test is made.

It has seemed to the writer that many cases affected with ulcerative conditions caused by other diseases than syphilis had stronger positive reactions than one would be led to expect from the amount of syphilitic anti-bodies that person ought to have, deducing from clinical evidence exhibited by the patient.

Hemolysis is the great factor in the causation of anemia. So we find that septicemia of different organisms, such as the

Typhoid Bacillus and streptococcus produce anemic conditions from the destruction of red blood cells by their toxins.

The toxin produced by carcinoma is known to be markedly hemolytic, therefore we get the cachexia of a long standing and uninterfered-with cancer. The anemia and hematuria of malaria is, no doubt, caused by the breaking down of red cells by the plasmodia and their toxins.

The venom of snakes, and the serum of snakes and eels is poisonous to the human because of their hemolytic properties.

The blood plasma contains fibrinogen, a globulin which, when under the influence of a fibrin ferment, becomes fibrin, an insoluble compound. Then there are euglobulins, serum-albumens, and a water soluble pseudoglobulin, albumoses, fats, sugars, and enzymes of various kinds which are also in the plasma.

Blood serum differs from blood plasma in that the fibrinogen is converted into fibrin by fribrin ferment.

This fibrin ferment is supposed to be in leukocytes and perhaps blood platelets, so there is liable to be more danger of thrombus or clot formation in cases with high leukocytosis and where the leukocytes are destroyed, as in Pneumonia and some of the pus-producing conditions.

Injury to the leukocytes by trauma also brings on coagulation.

If calcium is taken out of the blood, the blood does not coagulate, conversely it is a well known clinical fact that calcium given by the mouth increases its coagulability.

Alkalinity of the blood is important for its bactericidal power which increases with its alkalinity.

Blood that is rich in red blood cells is more alkaline as they contain the diffusable alkaline carbonates and phosphates. Venous blood is more alkaline than arterial blood.

Therefore the person whose red blood cell count is high, with a good color index, offers good resistance to disease.

The value of hyperemia and counter irritation is then shown in the treatment of local infection.

Those who do autopsies note that the right side of the heart is generally less damaged than the left by infection, due in some way perhaps to increased alkalinity and therefore bacteriacidal power of venous blood.

An herbivorous animal fed an excess of Hydrochloric acid becomes very sick and soon dies from symptoms of asphyxiation.

The power of the blood to carry carbondioxide is lessened and so it is not taken up from the tissues nor replaced by oxygen: so we get oxygen starvation of the tissues, and the patient has the symptoms, in the later stages, of acidosis, of asphyxiation, unconsciousness and coma.

If the alkalinity is not interfered with to the extent of producing asphyxiation of the tissues, the bacteriacidal power of the blood is lost and the patient is prone to infection. The meat eater is safer in this respect than the vegetarian is, for the end products of meat eating are nitrogen products; nitrogen makes ammonia and the ammonia neutralizes the acidosis.

In the time to which this paper must be limited we cannot treat of all sides of the broad subject of this fluid, the still mysterious fluid, the life of the system.

In spite of our so-called modern knowledge and enlightenment, if we bleed to the point where the blood pressure is too low for the blood to functionate, we die.

Men say bravely but mistakenly that they are not frightened by the sight of blood, for the average man is affected by the sight of his own blood, and many of them strong men, to the point of fainting if they are not in the prone position, so that the writer never takes blood for a blood culture or a Wassermann test unless the patient is lying down.

Science does not explain that, nor why a respectable member of bovine society loses her matronly dignity and acts the fool at the sight of blood.

Ehrlich speaks of his side-chain theory to express a belief he has not fully proven. So we make diagramatic representations of complements, amboceptors, toxophores, heptaphores, ceptors and receptors, and because the Germans would not recognize the work of Bordet and Gay, who were the originators of the theory and also of complement fixation test, these men called them lysins and thermolable and thermostable substances.

So the investigators investigate, describe and analyze and wonder over the phenomena of the blood. The more they study the more they analyze, the more are they convinced that they must agree with the chroniclers of ancient time that "The Blood is the Life."

Candler Building, Atlanta, Ga.

